# Septic perimyocarditis due to a right‐sided infective endocarditis of atypical morphology in a 33‐year‐old woman

**DOI:** 10.1002/ccr3.2914

**Published:** 2020-05-05

**Authors:** Jan‐Per Wenzel, Benedikt Schrage, Christoph Sinning, Stefan Blankenberg, Elvin Zengin‐Sahm, Edith Lubos

**Affiliations:** ^1^ Department of General and Interventional Cardiology University Heart Center Hamburg Hamburg Germany

**Keywords:** atypical, echocardiography, endocarditis, infective, perimyocarditis, vegetation

## Abstract

Perimyocarditis should be considered in patients with endocarditis not improving with conventional therapy and without typical valvular abnormalities. Vegetations can be sited anywhere in the atrium or ventricle and exhibit multiple shapes.

## Graphical abstract

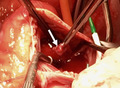

## 1 SUMMARY: HERE THE ABSTRACT SUMMARIZES THE CASE

A 33‐year‐old‐woman was admitted because of fever and increasing fatigue. Initial transthoracic echocardiography was inconclusive. Transesophageal echocardiography showed a tubular structure in the right atrium. Despite antibiotic therapy, the patient's condition worsened significantly. Intraoperatively, a perimyocarditis was diagnosed. After surgical excision and mechanical heart support, the patient stabilized.

## 2 PATIENT PRESENTATION

A 33‐year‐old woman was admitted to an external hospital because of fever and increasing fatigue. She had a history of intravenous drug abuse with a chronic hepatitis C and was diagnosed with bilateral pneumonia with pleural effusion and acute kidney failure 1 month before admission. No medication was taken previously. Except a subfebrile body temperature, the physical examination was unobtrusive with normal blood pressure and pulse, physiological auscultation of the lung and heart as well as a soft abdomen. No specific clinical signs of infective endocarditis (IE) were visible.

## 3 INITIAL WORKUP

**FIGURE 1:**
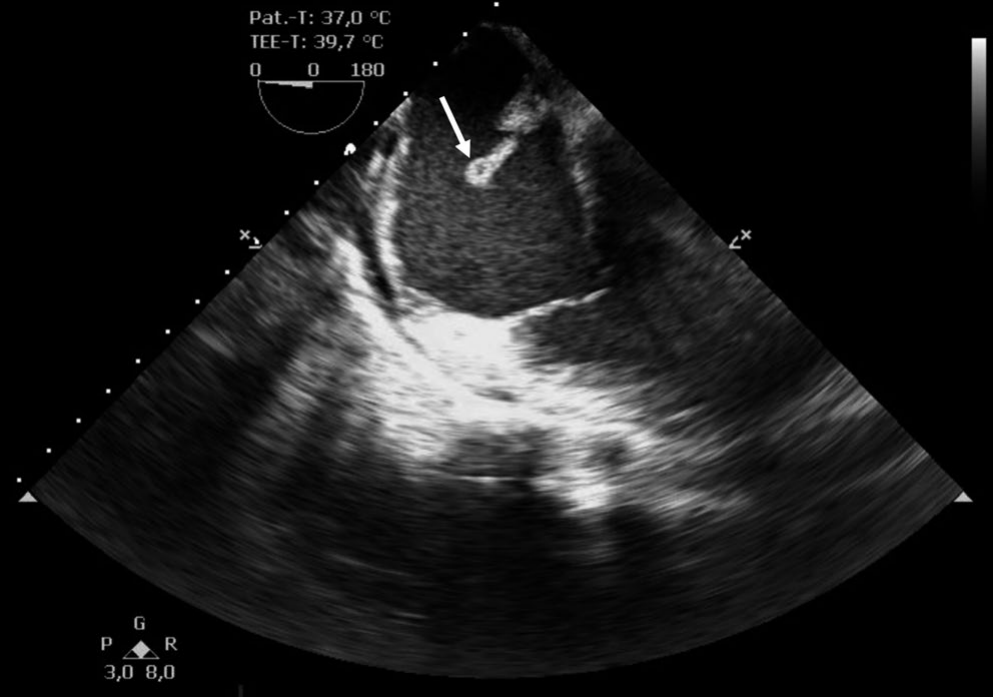
Transesophageal echocardiography: modified midesophageal four‐chamber view of the mobile, tubular structure in the right atrium. In the modified midesophageal four‐chamber view, an echogenic, mobile, tubular structure in the right atrium was visible. No involvement of the tricuspid valve was apparent. An additional movie file is included, underlining both the atypical, tubular structure and the unchanged tricuspid valve (see Video S1)

**FIGURE 2:**
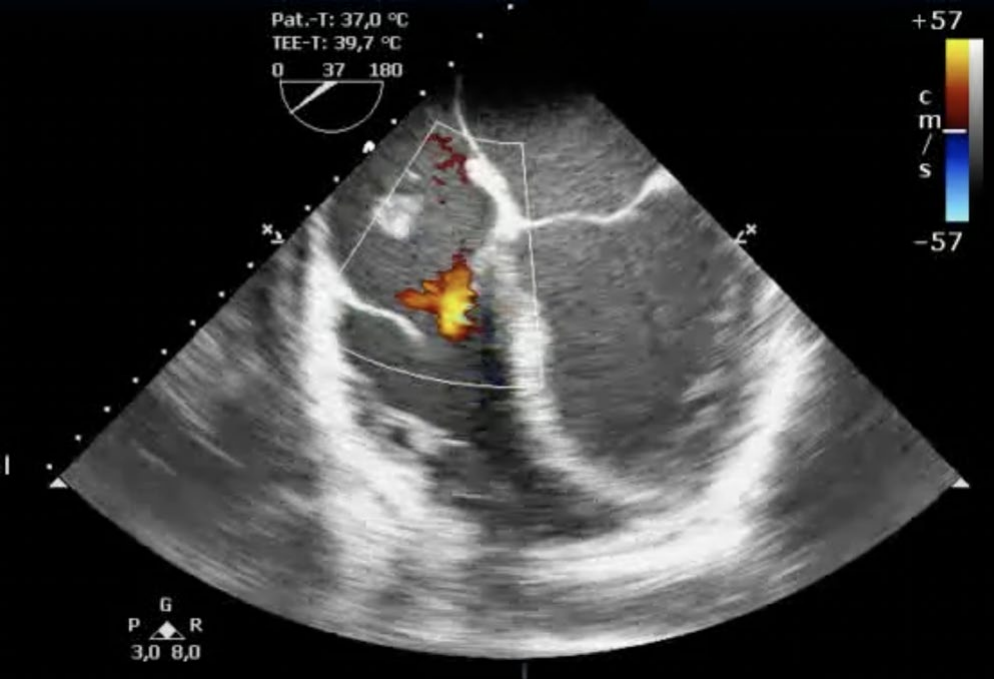
Transesophageal echocardiography: midesophageal four‐chamber view with no relevant regurgitation of the tricuspid valve. Color Doppler of the tricuspid valve in the midesophageal four‐chamber view revealed no relevant regurgitation. The tubular structure was partially visible in the right atrium. An additional movie file is included, displaying the integrity of the tricuspid valve assessed by color Doppler (see Video S2)

**FIGURE 3:**
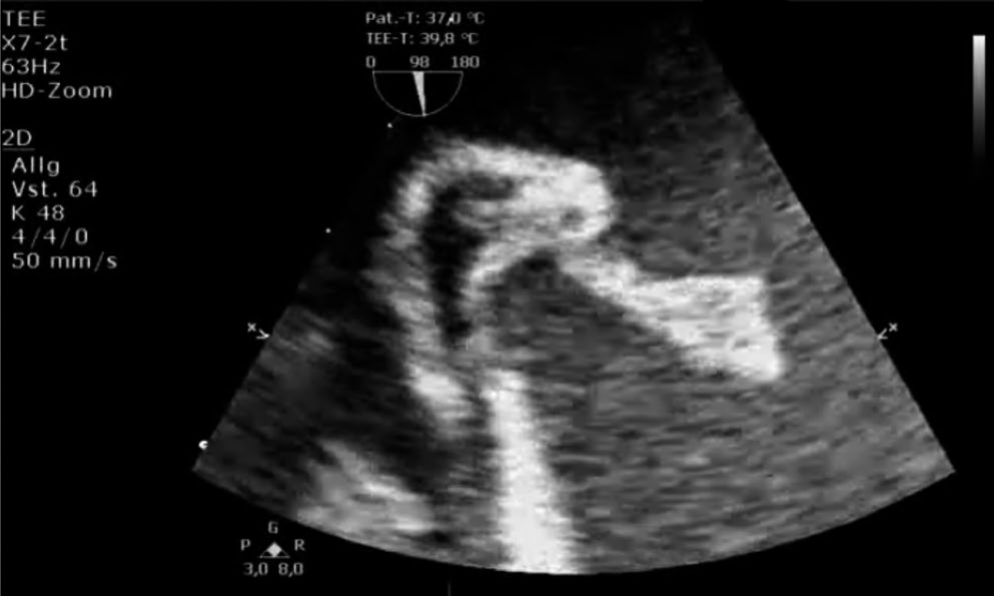
Transesophageal echocardiography: focussed midesophageal short‐axis view of the mobile, tubular structure in the right atrium. A focussed midesophageal short‐axis view of the right atrium showed the echogenic, mobile, tubular structure at the transition of the inferior vena cava into the right atrium. It measured 4.1 cm. Although being of relative high echogenicity and atypical location, in the context of positive blood cultures and intravenous drug abuse, it was highly suspect of a vegetation. An additional movie file is included, showing the movement of the tubular structure (see Video S3)

**FIGURE 4:**
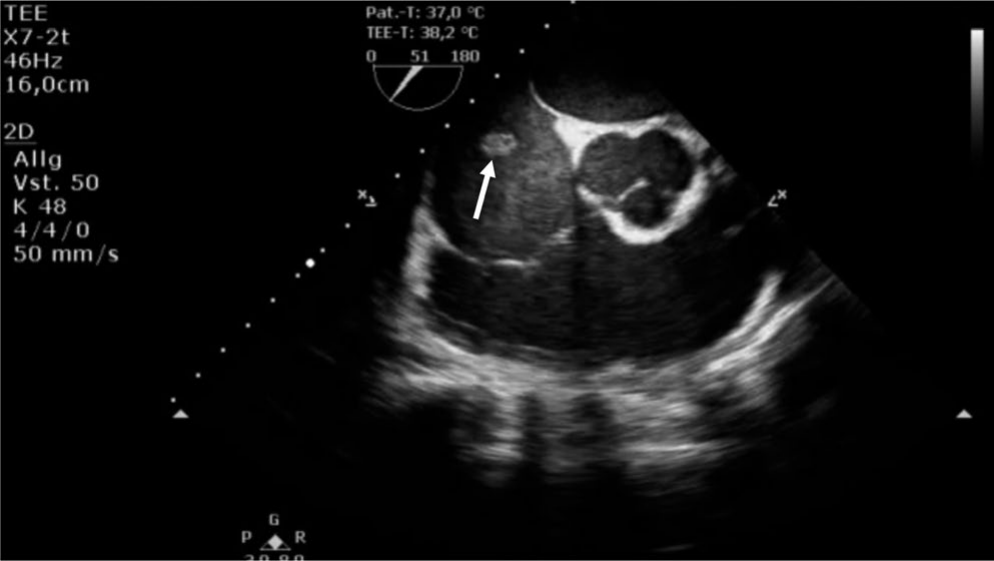
Transesophageal echocardiography: midesophageal short‐axis view showed no involvement of the right‐sided valves. In midesophageal short‐axis view, neither the tricuspid or pulmonic valve nor the aortic valve showed involvement. An additional movie file is included and shows this more precisely (see Video S4)

The initial electrocardiogram (ECG) showed a normo‐frequent sinus rhythm without any pathological findings. In the laboratory analyses, the inflammation parameters were elevated (leukocytes 19, 14 × 10^9^/L; C‐reactive protein [CRP] 187 mg/L; procalcitonin [PCT] 3.9 ng/mL). In chest X‐ray, no pulmonary infiltrates were visible. Two separate blood cultures were positive for methicillin‐susceptible *Staphylococcus aureus* (MSSA). Transthoracic echocardiography (TTE) revealed a discrete, hemodynamically irrelevant pericardial effusion, measuring 0.71 cm during end‐diastole in front of the right ventricle, and a faintly visible, mobile, floating structure in the right atrium with inexplicit involvement of the tricuspid valve. We decided to conduct transesophageal echocardiography (TOE). Here, the mobile structure, measuring 4.1 cm, was of tubular morphology and placed at the junction of the inferior vena cava into the right atrium (Figures 1, 2, 3, 4, Videos [Link], [Link], [Link], [Link]). Although the structure was of relative high echogenicity and atypically localized, in the context of positive blood cultures and intravenous (IV) drug abuse, it was highly suspect of a vegetation. No involvement of either the right‐sided or the left‐sided valves was seen.

## 4 DIAGNOSIS AND MANAGEMENT

Taking into account two positive major Duke criteria (blood cultures positive with *S aureus* and imaging positive for IE), a right‐sided IE was diagnosed. The initial antibiotic therapy with flucloxacillin, gentamicin, and amoxicillin was de‐escalated to flucloxacillin targeting the MSSA. However, the patient's condition worsened significantly. Because of an acute kidney failure with anuria, dialysis therapy was initiated and the patient was transferred to our institution.

Upon presentation, the patient was hemodynamically unstable, infective parameters were persistently high and despite intensive care treatment, and no clinical stabilization could be reached. After discussing the case in our interdisciplinary Endocarditis Team and considering other etiologies (eg, infected thrombus and malignant endocardial neoplasm), we decided to perform urgent surgery. The most common indication for surgery in IE is heart failure.^1^ Locally uncontrolled infection (eg, abscess and fistula) or persistent infection and the prevention of embolism are further indications for surgery.^2^ Vegetations with a length of >10 mm show a higher risk of embolism, with an even larger risk for larger (>15 mm) and mobile vegetations, especially caused by *Staphylococcus*.^3^, ^4^

**FIGURE 5:**
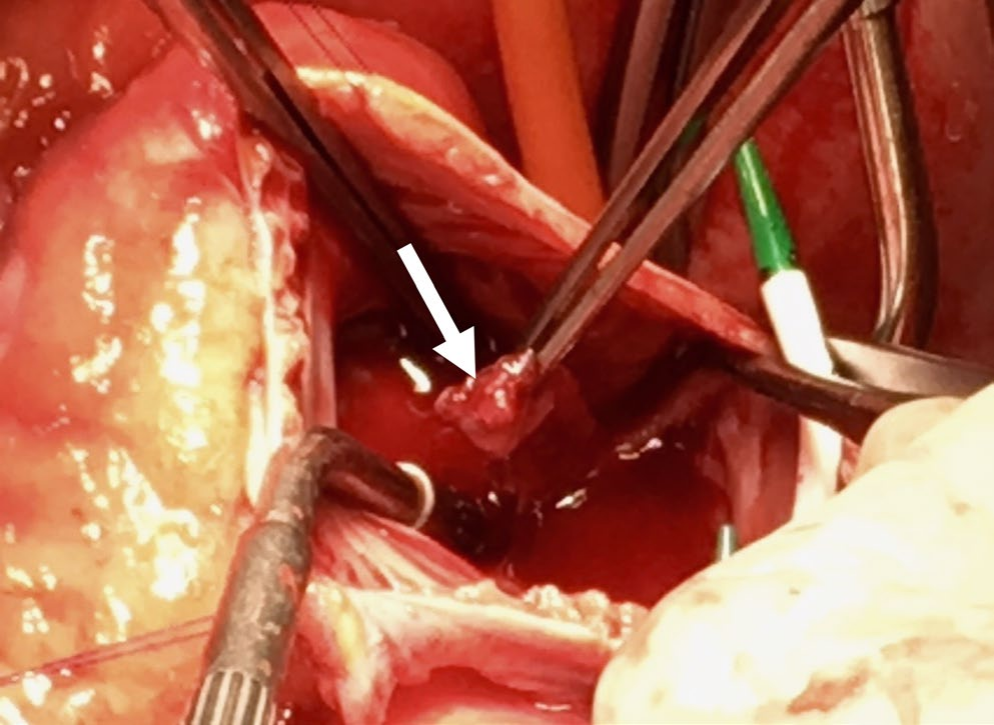
Surgical excision of the atypical vegetation in the right atrium. Intraoperative view of the tubular, atypical vegetation in the right atrium before excision

Intraoperatively, a septic perimyocarditis was observed. We excised the tubular structure in the right atrium (Figure 5) between the inferior vena cava and the coronary sinus, took a myocardial biopsy of the right ventricle (RV), and collected pericardial effusion for microbiological analysis. Furthermore, we implanted a femoral veno‐arterial extracorporeal membrane oxygenation system as a bridging device because of severely impaired biventricular function (LVEF [left ventricular ejection fraction] <15%; TAPSE [tricuspid annular peak systolic excursion] <8 mm).

## 5 FOLLOW‐UP

Histopathology from RV biopsy showed an invasion of the myocardium with CD3‐positive T‐lymphocytes, CD68‐positive macrophages, and neutrophil granulocytes combined with degeneration of adjacent myocytes consistent with active endomyocarditis. After continuous improvement of clinical and laboratory parameters as well as complete normalization of the cardiac systolic function, the patient was discharged from the intensive care unit after 7 days and referred to ambulatory care after 12 days. There was no residuum from this episode upon discharge.

## 6 CONCLUSION

With approximately 5% of all IE, right‐sided IE is a fairly rare disease.^5^ The most important risk factors are intravenous drug abuse and implanted foreign material. *S aureus* is still most prominent in IE.^6^ Transthoracic echocardiography can be challenging for diagnosing right‐sided IE due to image quality limitations. TOE is the technique of choice and should be considered in all patients with suspected IE.^2^ In the vast majority of cases, the tricuspid valve is involved.^7^, ^8^ In particular, findings of atypical location and morphology, as in our case, can be indicative for a perimyocarditis and complicate the diagnosis. Interdisciplinary discussion in an Endocarditis Team is of high importance, particularly in the context of surgical treatment, and fundamentally improves the outcome.

## CONFLICT OF INTEREST

None to declare.

## AUTHOR CONTRIBUTIONS

J‐PW: contributed to writing of the case study and data acquisition. BS: contributed to involvement in the writing process. CS: contributed to involvement in the writing process. SB: contributed to involvement in the writing process. EZ‐S: contributed to involvement in the diagnosing the disease and treatment of the patient. EL: contributed to involvement in the writing process as well as therapeutic decision.

## Supporting information

Video S1

Video S2

Video S3

Video S4
